# Frying stability of canola oil supplemented with ultrasound‐assisted extraction of *Teucrium polium*

**DOI:** 10.1002/fsn3.1405

**Published:** 2020-01-20

**Authors:** Yegane Asadi, Reza Farahmandfar

**Affiliations:** ^1^ Department of Food Science & Technology Sari Agricultural Sciences & Natural Resources University Sari Iran

**Keywords:** canola oil, frying, stability, *Teucrium polium*

## Abstract

In this study, antioxidant activity and protective effects of *Teucrium polium* extract in stabilizing of canola oil during frying were tested and compared to synthetic antioxidant, BHA. Total phenolic, α‐tocopherol, flavonoid, and condensed tannin content of *Teucrium polium* extracted by ethanol ultrasound‐assisted were 60.90 mg/g, 103.66 μg/ml, 4.36 mg/g, and 3.77 mg/g, respectively. Moreover, IC_50_ of the extract was 924.21 ppm. Canola oil samples containing 200, 600, and 1,000 ppm of the extract were heated at 180˚C for 30 hr and compared with BHA. Progress of oil oxidation was determined by measuring their peroxide value (PV), acid value (AV), iodine value (IV), carbonyl value (CV), color index (CI), conjugated diene value, and total polar compounds (TPC). The results showed that the extract was capable of retarding oil oxidation and deterioration significantly (*p* < .05) at all concentrations during frying. So, *Teucrium polium* extract can be used as natural antioxidant to retard oil oxidation.

## Graphical abstract

Canola oil formulated with *Teucrium polium* extract showed the lower degradation of oil oxidation products during frying. Therefore, *Teucrium polium* extract can be used as natural antioxidant to stability and safety of canola oil during frying.

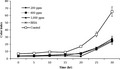

## 1 INTRODUCTION

Deep‐fat frying is a one of the oldest and popular food preparations. Frying is process of immersing food in hot oil with contact among oil, air, and food at high temperature. The frying process is influenced by the level of chemical reactions like hydrolysis, oxidation, and polymerization, which are interrelated and produce changes in chemical and physical characteristics features of frying oils (Choe & Min, 2007). The oxidation changes the nutritional quality of fat and oils (Farahmandfar, Asnaashari, & Sayyad, 2015). Due to these changes, consumers do not accept oxidized products and industries suffer from economic loss (Asnaashari, Farhoosh, & Farahmandfar, 2019; Iqbal & Bhanger, 2007).

Vegetable oils with high contents of unsaturated fatty acids, especially polyunsaturated fatty acids (PUFA), are more susceptible to oxidation (Mohdaly, Smetanska, Ramadan, Sarhan, & Mahmoud, 2011). Canola oil contains large amounts of omega‐3 fatty acids and it is good source of tocopherol contrasted with the other vegetable oils. Studies showed that canola oil, with high amount of monounsaturated and polyunsaturated fatty acids, reduces the levels of LDL cholesterol in the blood (Daun, Eskin, & Hickling, 2015). Due to the high content of unsaturated fatty acids in canola oil, the stabilizing of this oil against oxidation is important (Yassari & Yasari, 2013). Synthetic antioxidants such as butylated hydroxyl toluene (BHT), butylated hydroxyanisole (BHA), and tert‐butylhydroquinone (TBHQ) are often added to oils to retard oxidative degradation during frying (Asnaashari, Farhoosh, & Farahmandfar, 2016; Iqbal & Bhanger). However, BHA and BHT applications in frying are limited due to their decomposition or volatilization (Asnaashari, Farhoosh, & Sharif, 2014). In addition, these synthetic antioxidants are known to have toxic and carcinogenic effect on humans (Sayyari & Farahmandfar, 2017). Synthetic antioxidants may cause liver swelling and influence liver enzyme activities (Habib & Shah, 2004). Hence, there is a tendency towards the use of natural antioxidants to replace these synthetic antioxidants (Asnaashari, Hashemi, Mohammad, Mehr, & Asadi Yousefabad, 2015; Mohdaly et al., 2011).

Plant extract were found as source of antioxidant due to their high contents of phenolic compounds (Farahmandfar, Safari, Ahmadi Vavsari, & Bakhshandeh, 2015; Farahmandfar, Tirgarian, Dehghan, & Nemati, 2019). Therefore, they are widely used to retard lipid oxidation in foods. The use of ultrasonic instruments for extraction purpose in raw materials is considered as an economical alternative to traditional extraction process, this being a demand by industry for sustainable development. Ultrasound assistance has already demonstrated an important effect on the extraction of phenolic from other vegetable sources (d’Alessandro, Kriaa, Nikov, & Dimitrov, 2012). The mechanical effect of ultrasound is attributed to increasing of mass transfer arising from the collapse of cavitation bubbles near cell walls. Ultrasound can break down the cell walls and promote better penetration of solvent into plant cells (Albu, Joyce, Paniwnyk, Lorimer, & Mason, 2004).

*Teucrium polium* is a plant which belongs to the Lamiaceae. This family is composed of species with antioxidant potential. *Teucrium polium* is one of the wild‐growing flowering species from this genus and is found abundantly in Mediterranean like Iran (Sharififar, Dehghn‐Nudeh, & Mirtajaldini, 2009; Tepe, Degerli, Arslan, Malatyali, & Sarikurkcu, 2011). This plant is used to prepare herbal tea and traditional medicine. The tea of *Teucrium polium* is used as an appetizer especially in children and also as a spice. An infusion of the leaves and flowers of the plant is consumed as a refreshing beverage. The biological activities of *Teucrium polium* are widely reported, and it has been shown to possess anti‐inflammatory, antinociceptive, antibacterial, antihypertensive, hypolipidemic, antirheumatoid, and hypoglycemic effects (Hasani et al., 2007). The therapeutic benefit of medicinal plants like *Teucrium polium* was attributed to their antioxidant properties (Sharififar et al., 2009). There are also some reports in the literature for antioxidant effects of crude extract of *Teucrium polium* (Hasani et al., 2007; Ljubuncic et al., 2006).

The aim of the presented study was to characterize the effects of different concentrations of ethanol ultrasound‐assisted of *Teucrium polium* extract on the oxidative stability of canola oil during frying and compare with BHA.

## 2 MATERIALS AND METHODS

### 2.1 Materials

*Teucrium polium* (Halpeh) plant was collected from Shiraz city, Fars Province, Iran, in 2015. Refined canola oil, without added antioxidants, was obtained by Damoon Company. All chemical materials and solvents were of analytical grade and bought from Sigma‐Aldrich or Merck.

### 2.2 Ultrasound‐assisted extraction

Twenty grams of *Teucrium polium* leaves powder was mixed with 100 ml of ethanol‐aqueous (50:50) solvent and sonicated for 45 min in an ultrasonic bath (20 kHz Frequency). After three stages of centrifuge (Hermie z200 A‐Germany, 3,000 rpm for 10 min), the supernatant was filtered through Whatman filter paper No. 1. Then, the solution was completely evaporated in an oven (45˚C, 3 hr). The residual crude of extract was covered and stored at −18°C.

### 2.3 Chemical compounds of extract

#### 2.3.1 Determination of total phenolic content

The amount of phenolic compounds in the extracts was determined with Folin–Ciocalteu regent following the colorimetric method (Pourmorad, Hosseinimehr, & Shahabimajd, 2006) with some modifications. Methanolic solutions of *Teucrium polium* extracts (1 mg/ml) were prepared for the analysis. Briefly, 0.5 ml methanolic extract solution was mixed with 2.5 ml of 10% Folin–Ciocalteu reagent dissolved in distilled water and 2 ml of sodium carbonate solution (7.5%). The blank contained 0.5 ml methanol, 2.5 ml Folin–Ciocalteu reagent (10%), and 2 ml of 7.5% sodium carbonate solution. Then, the mixture was incubated at room temperature for 30 min for the development of a blue color. The absorbance was measured at 765 nm with a UV‐vis spectrophotometer. A similar procedure was carried out for gallic acid standard solution, and the calibration curve was prepared from various concentrations of gallic acid. The total phenolic content was expressed as mg/g.

#### 2.3.2 Determination of total α‐tocopherol content

The total amount of tocopherol components of the extracts was estimated based on α‐tocopherol. In this test, 200 mg of the extract was mixed with 5 ml of toluene, and then, 3.5 ml of 2,2 bipyridine solution and 0.5 ml iron chloride III was added and mixed, and finally, the volume of solution with ethanol (95%) was raised to 10 ml and placed unmoved for 1 min and its absorbance was read at 520 nm using a spectrophotometer (Wong, Timms, & Goh, 1988).

#### 2.3.3 Determination of total flavonoid content

A volume (5 ml) of 2% AlCl_3_ in methanol was blended with 5 ml of the extract solution (0.4 mg/ml). Absorbance was read at 415 nm by UV‐vis spectrophotometer after 10 min against a blank sample (5 ml extract solution with 5 ml methanol without AlCl_3_). The total flavonoid content (mg/g) was measured by standard curve of catechin at the concentration of 0–100 mg/g (Ramamoorthy & Bono, 2007).

#### 2.3.4 Determination of total condensed tannin content

Four hundred microliters of extract was added to 3 ml of a vanillin solution (4% in methanol) and 1.5 ml of concentrated hydrochloric acid. After 15 min of incubation, the absorbance was read at 500 nm. Total condensed tannin was expressed as mg/g (Rebaya et al., 2014).

### 2.4 Estimating antioxidant activity by DPPH method

DPPH is a purple component which has turned into a radical due to the existence of phenyl group in its structure and is the free radical recourse. The scavenging capacity of DPPH free radicals was evaluated. In this study, 50 μl extract with concentration of 200, 400, 600, 800, 1,000, and 1,200 ppm was added to 5 ml of 0.004% DPPH in methanol and put in room temperature for 30 min, and then, light absorbance of samples was read at 517 nm. The DPPH free radical inhibition percentage (*I*%) was estimated according to the following formula, where *A*_blank_ is the absorbance of the control reaction (containing all reagents except the test compound) and *A*_sample_ is the absorbance of the test compound.

(1) $$I\%=\left[\left(A_{\text{blank}}-A_{\text{sample}}\right)/A_{\text{blank}}\right]\times 100$$

### 2.5 Frying condition

Potatoes were peeled and cut into pieces (7.0 × 0.5 × 0.3 cm) and submerged in water until needed. Potato pieces were fried in the frying oils. The *Teucrium polium* extract was added to canola oil (without additives) at three concentration levels including 200, 600 and 1,000 ppm and BHA (200 ppm), and the oils were placed in a 2.5 L capacity stainless steel fryer (Tefal Azura‐French) and heated at 180˚C for 30 hr. At the end of each 5 hr, about 10 g of the frying oil was filtered into a screw‐cap vial and quickly stored in the dark at 4˚C until analyzed. The volume of oil was not replenished during the frying process. Frying experiments were conducted in duplicate on each frying medium. The fryer was turned off at the end of the frying experiment and oil cooled to 60˚C before filtering and using separate filters to remove debris. Oxidation process was determined by oil stability index, acid value, peroxide value, carbonyl value, total polar compound, iodine value, conjugated dienes, and color index.

### 2.6 Determination of oil stability index (OSI)

Rancimat (Metrohm model 734, Herisan Switzerland) was used to measure the oil stability index of oil samples. A dry and clean current of air at the rate of 15 L/hr was passed through each oil sample. The air carrying volatile organic acids produced by the oxidation of the oil sample was guided into a container for measuring electrical conductivity to which 60 ml of distilled water had been added. Three grams of oil sample was needed for each test. The oil stability index was automatically measured at 120˚C (Farahmandfar, Asnaashari, et al., 2015).

### 2.7 Determination of acid value

The free fatty acids (FFAs) was evaluated consistent with the Official Method Cd 3d‐63 of AOCS (1993). Ten grams of each sample was put into an Erlenmeyer flask, and 50 ml of solvent ethanol:chloroform was added (the solvent must be neutral). Phenolphthalein was used as indicator, and then, each oil solution was titrated with the 0.01 N KOH solutions.

### 2.8 Determination of peroxide value

The PV was determined according to the AOCS (1993). In this method, 5 g of oil sample was weight in Erlenmeyer flask; then, 30 ml of acetic acid glacial–chloroform (3:2) solvent was added and stirred. Then, 0.5 ml of saturated solution of iodide potassium was added. The mixture was put in the dark place for 1 min. After added 30 ml of distilled water and 1 ml of starch indicator, the solvent was titrated with the sodium thiosulfate 0.01 N solution.

### 2.9 Determination of carbonyl value

First purification of solvents was done. To 1,000 g of solvent (2‐propanol), 0.6 g of NaBH_4_ (sodium borohydride) was added. Subsequently, this solution was refluxed for 30 min. This method eliminates carbonyl compounds present in solvent. 2,4‐Dinitrophenylhydrazine (2,4‐DNPH) solution was prepared by dissolving 50 mg DNPH in 100 ml solvent containing 3.5 ml concentrated HCL. Oil sample (0.16–1 g) was filled to the mark with the solvent. Standard aldehydes (2,4 decadienal and 2‐decenal) were weighed in a volumetric flask and dissolved in the solvent to reach concentration of 60–600 μmol. One milliliter of oil solution or standard carbonyl compound solution was poured into a 15‐ml test tube and then blended with 2,4‐DNPH solution (1 ml). At that time, the test tube was warmed (20 min, 40˚C). The test tube chilled for 10 min and 8 ml of potassium hydroxide solution (2%) was inserted and later, centrifuged at 4,600 rpm (5 min, 20˚C). Supernatant was removed, and the absorbance was determined by UV‐vis spectrophotometer at 420 nm (Farhoosh & Moosavi, 2006).

### 2.10 Determination of total polar compound

Silica gel 60 (70–230 mesh) was dried (12 hr, 160˚C), added to water (5:95), and stirred dynamically for about 1 min and remain overnight. At that moment, 1 g of the silica gel 60 was compressed and filled between two cotton wool balls into a 5‐ml pipette tip. Oil sample (500 mg) was pipette into a volumetric flask (5 ml). It was dissolved in toluene (4 ml) and then filled with the toluene. Next, the solution (1 ml) was pipette on top of the pipette tip. After the solution was soaked in, the pipette tip was washed with eluent (1 ml) and, after soaking in, was added with 7 ml of eluent. After 15 min elution, the end of the tip was cleaned with toluene (500 μl). After the solvent was removed, the TPC (%) was calculated using the following equation:

(2) $$\text{TPC}\left(\%\right)=\left(\frac{w-w_{1}}{w}\right)\times 100$$

which *w* is the sample weight (mg) and *w*_1_ is the weight of nonpolar compounds (mg) (Schulte, 2004).

### 2.11 Determination of iodine value

Iodine value (g/100g) of oil samples was determined by Hanus method (Horowitz & Latimer, 2006). 0.4 g of oil sample with chloroform (20 ml) was introduced into iodine flask and mixed to dissolved. At that time, Hanus solution (20–25 ml) was added and the flask was completely closed with parafilm and hold in dark (30 min). Then, 20 ml of saturated potassium iodide (15%) and distilled water (100 ml) was inserted and titrated with 0.1 N sodium thiosulfate solution up to yellow color created. At that point, 2 or 3 drops of starch solution were added and titrated until the blue color is formed.

### 2.12 Determination of conjugated dienes

The oil sample was diluted (1:600) with hexane, and their absorbance was read at 243 nm. Absorbance of the hexane was measured as blank (Saguy, Shani, Weinberg, & Garti, 1996).

### 2.13 Determination of color index

Absorption at 420 nm was determined on a spectrophotometer and read against water as blank (Saguy et al., 1996).

### 2.14 Statistical analysis

All measurements were performed in triplicate. Experimental data were analyzed using analysis of variance (ANOVA). Different among means (*p* < .05) were verified by Duncan's multiple range test using statistical system (SAS).

## 3 RESULTS AND DISCUSSION

### 3.1 Properties of *Teucrium polium* extract

#### 3.1.1 Extract yield, total phenolic, α‐tocopherol, flavonoid, and condensed tannin content

In this study, the yield of extraction (25.01%) was high which shows the economic value of this product. Phenolic compounds are widely distributed in the plant kingdom. There is a positive relation between total phenolic content and antioxidant activity in many plants. These compounds are known as high level antioxidants because of their ability to donate hydrogen atom and act as reducing agent and singlet oxygen quenches. Several oil seed and spices have been investigated for phenolic compounds in search for safe sources of natural antioxidant (Farahmandfar, Naeli, Naeli, Naderi, & Asnaashari, 2019; Mohdaly et al., 2011). The composition and phenolic content of fruit and vegetable were influenced by environmental and genetic factors as well as postharvest processing conditions (Asnaashari, Tajik, & Khodaparast, 2015). The results showed that the amount of phenolic compounds of *Teucrium polium* extracted by ethanol ultrasound‐assisted is 60.90 mg/g. This is in agreement with the result of researchers (Stankovic, Niciforovic, Mihailovic, Topuzovic, & Solujic, 2012) who study on phenolic content of different part of *Teucrium polium* extracted with different solvent. The total phenolic content of the *Teucrium polium* extract was higher than many plants: 2.28 mg/g for Bene Hull (Shaddel et al., 2014), 28.42–124.27 mg/g for marigold (*Tagetes erecta* L.) flower residues (Xu, Wang, Jiang, Yuan, & Gao, 2015), and 37.52 mg/g for XiLan olive fruit dreg (Yu, Zhu, Zhong, Li, & Ma, 2015).

α‐tochopherol is a lipid‐soluble antioxidant, and in green plant tissues, it is localized in the chloroplast envelope and thylakoid membranes (Brewer, 2011). Tocopherols react with lipid and peroxy radicals and change them to stabilized products. Tocopherols terminate the free radicals by donating a hydrogen atom to lipid peroxy radicals and produce lipid derivatives and antioxidant radicals that are more stable and less readily available to contribute in propagation reactions (Colombo, 2010). The concentration of α‐tochopherol in extract was found to 103.66 μg/ml in our study.

Because antioxidant activity does not always correlate with the polyphenolic compounds, flavonoids need to examine. Total flavonoid content of *Teucrium polium* extract was obtained 4.36 mg/g. Chemically, condensed tannins are defined as polymeric flavonoids. However, they can appear as oligomers as well, when they are composed of two to ten monomeric units. In the form of polymeric flavonoids, they have limited to no solubility in water, whereas in oligomeric form, they are water soluble. Within the flavonoids group, condensed tannins are considered as flavanols, since they are composed of flavan‐3‐ol moieties (de Hoyos‐Martínez, Merle, Labidi, & Charrier–El Bouhtoury, 2019). In this study, total condensed tannin content of *Teucrium polium* extract was obtained 3.77 mg/g.

#### 3.1.2 DPPH radical scavenging activity

The scavenging model of DPPH radical is widely used as a method for assessing antioxidant activity in a period relatively short compared to other method. DPPH is very stable organic free radical with deep violet color, which gives maximum absorption at the 515– 528 nm range. Upon receiving a proton from any hydrogen donor, mainly from phenolics, it loses color and become yellow. The scavenging of DPPH radical enhanced when amount of phenolic compounds or degree of hydroxylation of the phenolic compounds increased and our findings were in agreement with other studies (Jalali Mousavi, Niazmand, & Shahidi Noghabi, 2015; Sayyad & Farahmandfar, 2017). The IC_50_ calculated for extract was 924.21 ppm. Low IC_50_ corresponds to a strong inhibitory capacity of DPPH radical. Moreover, the extract yield of antioxidant compounds from *Teucrium polium* was 35.03%.

### 3.2 Properties of fried oil

#### 3.2.1 Oil stability index

Generally, oxidation carry on very slowly at the initial stage, the time to reach a rapid increase in oxidation rate is known as OSI or the induction period (IP). OSI is common approach to determine in resistance to oxidative rancidity of edible oils (Nor, Mohamed, Idris, & Ismail, 2008). Table 1 reveals the OSI of *Teucrium polium* extract compared to BHA and control. Oil stability index decreased after each frying cycle. The herb extracts in different concentrations showed higher OSI than the control and BHA. Extend induction period for *Teucrium polium* extract than BHA may be due to volatility of BHA than phenol compounds at high temperature. Induction time after 30 hr of frying process was decreased in following order: *Teucrium polium* at 1,000 ppm > *Teucrium polium* at 600 ppm > *Teucrium polium* at 200 ppm > BHA at 200 ppm > control. Greater OSI of canola oil with higher content of the *Teucrium polium* extract could be explained by declining of oxidation rate; therefore, the generation of lower amount of primary and secondary products was occurred. Overall, further quick production of the primary and secondary products leads to lower IP (Farahmandfar, Naeli, et al., 2019).

**Table 1:** Oxidative stability index (h) of canola oil with different concentrations of *Teucrium polium* extract (ppm) during frying process at 180°C

	Time (h)						
	0	5	10	15	20	25	30
200 ppm	6.85 ± 0.18 ^Aa^	6.24 ± 0.06 ^Bb^	5.85 ± 0.06 ^Cc^	5.45 ± 0.12 ^Dc^	4.37 ± 0.08 ^Ec^	3.20 ± 0.10 ^Fb^	2.25 ± 0.10 ^Gb^
600 ppm	6.86 ± 0.16 ^Aa^	6.61 ± 0.07 ^Ab^	6.20 ± 0.06 ^Bb^	5.77 ± 0.13 ^Cb^	4.63 ± 0.08 ^Db^	3.39 ± 0.11 ^Eb^	2.38 ± 0.10 ^Fb^
1,000 ppm	6.25 ± 0.15 ^Ca^	7.18 ± 0.07 ^Aa^	6.73 ± 0.07 ^Ba^	6.27 ± 0.14 ^Ca^	5.03 ± 0.09 ^Da^	3.68 ± 0.11 ^Ea^	2.58 ± 0.11 ^Fa^
BHA	5.80 ± 0.14 ^Ab^	5.66 ± 0.06 ^Ac^	5.31 ± 0.05 ^Bd^	4.94 ± 0.11 ^Cd^	3.96 ± 0.07 ^Dd^	2.90 ± 0.09 ^Ec^	2.04 ± 0.09 ^Fc^
Control	4.47 ± 0.11 ^Ac^	4.36 ± 0.04 ^Ad^	4.08 ± 0.04 ^Be^	3.80 ± 0.08 ^Ce^	3.05 ± 0.05 ^De^	2.23 ± 0.07 ^Ed^	1.57 ± 0.07 ^Fd^

Note

Means ± standard error within a row with the same uppercase letters is not significantly different at *p* < .05. Means ± standard error within a column with the same lowercase letters is not significantly different at *p* < .05.

#### 3.2.2 Acid value

The hydrolysis of oil leads to the formation mono and di‐glycerids of fatty acids and glycerol. The degradation of secondary products of oxidation formed during heating or existing of moisture or oxygen from air effect on acidity and increase the FFAs value. These variations are dependent on the initial volume of this parameter and heating time (Eshghi, Asnaashari, Haddad Khodaparast, & Hosseini, 2014; Farahmandfar, Asnaashari, Pourshayegan, Maghsoudi, & Moniri, 2018). Hydrolysis is the reaction affecting edible oils due to the action of lipolitic enzymes or moisture, but this reaction does not happen during our study for the reason that heating was conducted in only canola oil without potato or other foods (Chammem et al., 2015). The AV content of all samples increased gradually from 0 until 30 hr of frying. The considerable increase was observed in 25 and 30 hr of frying (Table 2). The AV for control was significant (*p* < .05) higher than BHA and oil containing *Teucrium polium* extract. Result shows that *Teucrium polium* extract and BHA were capable of decreasing AV in frying canola oil. The lower AV for sample with the *Teucrium polium* extract than that in the control might be due to their higher antioxidant activity. The high amount of total phenolic compounds in the *Teucrium polium* extract may be responsible for the decrease AV in canola oil during frying.

**Table 2:** Acid value (%) of canola oil with different concentrations of *Teucrium polium* extract (ppm) during frying process at 180°C

	Time (h)						
	0	5	10	15	20	25	30
200 ppm	0.11 ± 0.02 ^Ca^	0.08 ± 0.02 ^Cb^	0.11 ± 0.02 ^Cb^	0.11 ± 0.02 ^Cb^	0.12 ± 0.02 ^Cb^	0.22 ± 0.03 ^Bb^	0.50 ± 0.02 ^Ab^
600 ppm	0.10 ± 0.02 ^Ca^	0.08 ± 0.02 ^Cb^	0.10 ± 0.02 ^Cc^	0.10 ± 0.02 ^Cb^	0.11 ± 0.02 ^Cb^	0.20 ± 0.02 ^Bb^	0.46 ± 0.02 ^Ac^
1,000 ppm	0.10 ± 0.02 ^Ca^	0.07 ± 0.02 ^Db^	0.09 ± 0.02 ^Cc^	0.10 ± 0.02 ^Cb^	0.10 ± 0.02 ^Cb^	0.19 ± 0.02 ^Bb^	0.44 ± 0.01 ^Ac^
BHA	0.09 ± 0.02 ^Ca^	0.07 ± 0.02 ^Cb^	0.09 ± 0.02 ^Cc^	0.09 ± 0.02 ^Cb^	0.09 ± 0.02 ^Cb^	0.17 ± 0.02 ^Bc^	0.39 ± 0.01 ^Ad^
Control	0.12 ± 0.02 ^Fa^	0.20 ± 0.04 ^Ea^	0.20 ± 0.04 ^Ea^	0.28 ± 0.04 ^Da^	0.40 ± 0.04 ^Ca^	0.77 ± 0.04 ^Ba^	1.05 ± 0.04 ^Aa^

Note

Means ± standard error within a row with the same uppercase letters is not significantly different at *p* < .05. Means ± standard error within a column with the same lowercase letters is not significantly different at *p* < .05.

#### 3.2.3 Peroxide value

The primary products of lipid oxidation are hydroperoxides, which are generally referred to as peroxides. The peroxide value is the classical method for verifying the degree of edible oil oxidation and measures hydroperoxides formation (Farhoosh, Sharif, Asnaashari, Johnny, & Molaahmadibahraseman, 2016; Senanayake, 2013). It is a way to measure the amount of primary oxidative products in oils. The influence of the extracts and BHA on PVs in canola oil is shown in Table 3. A continuous rise in peroxide value with the increase in frying time was perceived for the entire samples in the beginning. The PV for control was significant (*p* < .05) more than the oil containing different concentrations of extract and 200 ppm BHA. Due to the instability of peroxides, byproduct such as carbonyl and aldehydes are produced. In other words, decomposition of hydroperoxide caused declining trend in PV after 15 min, however the carbonyl increased. This behavior indicated that hydroperoxides are formed slower than being decomposed. The peroxide value of control oil sample, free of additives, reached to greatest value 6.60, while for 200, 600, and 1,000 ppm of *Teucrium polium* extract and 200 ppm of BHA were 3.24, 2.98, 2.81, and 2.55 meqO_2_/kg after 30 hr of frying, respectively. Higher concentrations of *Teucrium polium* extract are more effective in preventing peroxide formation. This can be associated to the increase in the antioxidant activities with phenolic compounds. The present study showed that increasing temperature accelerates the oxidation rate of canola oil but the addition of *Teucrium polium* extract to oil resulted in increased stability.

**Table 3:** Peroxide value (meqO_2_/kg) of canola oil with different concentrations of *Teucrium polium* extract (ppm) during frying process at 180°C

	Time (h)						
	0	5	10	15	20	25	30
200 ppm	0.47 ± 0.07 ^Db^	0.48 ± 0.07 ^Db^	0.54 ± 0.08 ^Cb^	0.56 ± 0.08 ^Cb^	0.50 ± 0.07 ^Cb^	0.68 ± 0.09 ^Bb^	3.24 ± 0.46 ^Ab^
600 ppm	0.43 ± 0.06 ^Dc^	0.44 ± 0.06 ^Db^	0.49 ± 0.07 ^Db^	0.51 ± 0.07 ^Cc^	0.46 ± 0.06 ^Dc^	0.63 ± 0.09 ^Bc^	2.98 ± 0.04 ^Ac^
1,000 ppm	0.41 ± 0.06 ^Cc^	0.42 ± 0.06 ^Cb^	0.47 ± 0.07 ^Cb^	0.48 ± 0.07 ^Cc^	0.43 ± 0.06 ^Cd^	0.59 ± 0.08 ^Bc^	2.81 ± 0.04 ^Ad^
BHA	0.37 ± 0.05 ^Cc^	0.38 ± 0.05 ^Cc^	0.42 ± 0.06 ^Cc^	0.44 ± 0.06 ^Cd^	0.39 ± 0.05 ^Cd^	0.54 ± 0.07 ^Bc^	2.55 ± 0.04 ^Ae^
Control	0.69 ± 0.10 ^Da^	0.75 ± 0.11 ^Da^	0.82 ± 0.12 ^Da^	0.83 ± 0.11 ^Da^	1.03 ± 0.15 ^Ca^	3.50 ± 0.50 ^Ba^	6.61 ± 0.92 ^Aa^

Note

Means ± standard error within a row with the same uppercase letters is not significantly different at *p* < .05. Means ± standard error within a column with the same lowercase letters is not significantly different at *p* < .05.

#### 3.2.4 Carbonyl value

Table 4 shows changes in carbonyl value of the canola oil during frying process. Determination of peroxide value is not suitable for the assessment of frying oil. Peroxide is unstable and hydroperoxide decomposition generates secondary oxidative products. Carbonyl index measures the secondary products of oxidation that were created from the decomposition of hydroperoxides and considered to be good indicator of oxidative change in edible oils. The carbonyl compounds in oils during frying are essential for determining the quality of edible oils. The CV in oils during frying promote the rancidity and off‐flavors and decrease nutritional compounds in fried foods (Farahmandfar, Asnaashari, et al., 2015; Farahmandfar, Naeli, et al., 2019; Sayyad & Farahmandfar, 2017). During the frying process of the canola oil, the CV increased until 15 hr for all of the samples, and then, a decreasing trend was observed. This was attributed to the decomposition of carbonyl compounds during the prolonged heating period and the formation of new compounds which were not detectable by the CV assay. The present result is in agreement with those reported in previous researchers (Farahmandfar, Asnaashari, et al., 2015; Farahmandfar, Naeli, et al., 2019; Farhoosh & Kenari, 2009). After 30 hr of frying, carbonyl value reached 31.65, 29.12, and 27.52 μmol/g for 200, 600, and 1,000 ppm of *Teucrium polium* extract, respectively. However, the carbonyl value of BHA and control sample was 24.93 and 64.48 μmol/g, respectively. Control samples show the highest carbonyl value, indicating that BHA and *Teucrium polium* extract can reduce formation of carbonyl compound during the frying process of the canola oil.

**Table 4:** Carbonyl value (µmol/g) of canola oil with different concentrations of *Teucrium polium* extract (ppm) during frying process at 180°C

	Time (h)						
	0	5	10	15	20	25	30
200 ppm	6.54 ± 0.40 ^Db^	6.84 ± 0.42 ^Db^	8.97 ± 0.66 ^Cb^	9.01 ± 0.69 ^Cb^	6.85 ± 0.42 ^Db^	19.21 ± 0.58 ^Bb^	31.65 ± 1.60 ^Ab^
600 ppm	6.02 ± 0.37 ^Db^	6.30 ± 0.39 ^Dc^	8.00 ± 0.61 ^Cc^	8.26 ± 0.63 ^Cc^	6.30 ± 0.39 ^Dc^	17.68 ± 0.54 ^Bc^	29.12 ± 1.47 ^Ac^
1,000 ppm	5.69 ± 0.35 ^Db^	5.95 ± 0.37 ^Dd^	7.56 ± 0.58 ^Cc^	7.83 ± 0.60 ^Cd^	5.95 ± 0.37 ^Dc^	16.70 ± 0.51 ^Bd^	27.52 ± 1.40 ^Ac^
BHA	5.28 ± 0.33 ^Eb^	5.39 ± 0.33 ^Ee^	6.85 ± 0.52 ^Dd^	7.10 ± 0.54 ^Ce^	5.40 ± 0.33 ^Ed^	15.14 ± 0.46 ^Be^	24.93 ± 1.27 ^Ad^
Control	9.79 ± 0.60 ^Ga^	10.77 ± 0.67 ^Fa^	13.24 ± 1.01 ^Ea^	23.52 ± 0.72 ^Ca^	16.95 ± 1.30 ^Da^	34.00 ± 2.37 ^Ba^	64.48 ± 3.26 ^Aa^

Note

Means ± standard error within a row with the same uppercase letters is not significantly different at *p* < .05. Means ± standard error within a column with the same lowercase letters is not significantly different at *p* < .05.

#### 3.2.5 Iodine value

**Figure 1:**
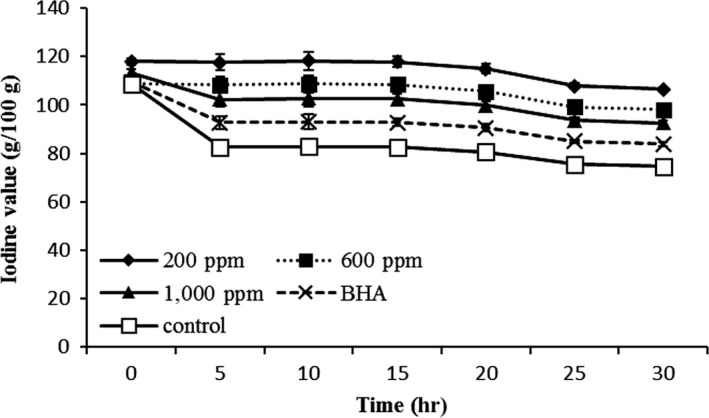
Changes in iodine value of canola oil with different concentrations of *Teucrium polium* extract (ppm) during frying process at 180°C

The IV is a measure of the degree of unsaturation of fatty acid. Heating oil during deep fat frying leads to rancidity and influences the double bounds of unsaturated fatty acids and results in the IV reduction. The highest decrease in double bounds occurred due to oxidative rancidity in the frying canola oil. This observation could be due to the presence of the high amount of PUFA especially linolenic acid in canola oil. Figure 1 shows the comparison of *Teucrium polium* extract with synthetic antioxidant and control samples in the term of iodine value during 30 hr of frying canola oil. Iodine value decreases gradually during frying. However, no regular pattern of decrease was observed. Result shows that IV of fried canola oil treated with synthetic antioxidant and *Teucrium polium* extract was definitely higher than control sample of canola oil. IV of canola oil treated with different concentration of *Teucrium polium* extract was higher than BHA at the end of 30 hr of frying, showing a better ability of *Teucrium polium* extract to prevent the reduction of IV than BHA.

#### 3.2.6 Conjugated diene

**Figure 2:**
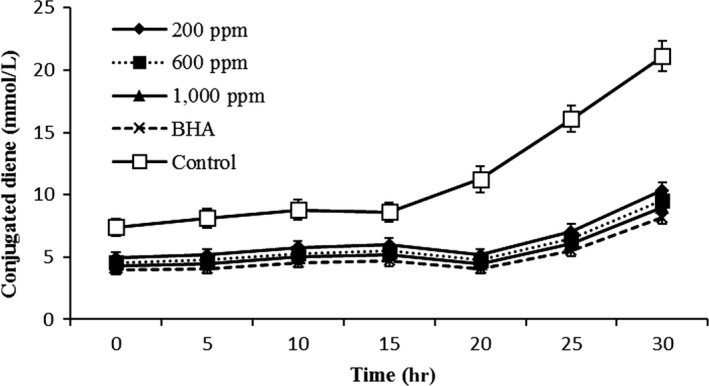
Changes in conjugated dienes of canola oil with different concentrations of *Teucrium polium* extract (ppm) during frying process at 180°C

Conjugated diene measures the first change during oxidation of oil rich in polyunsaturated fatty acids. Good correlation between CD and PV has been found. During lipid oxidation, double bonds in unsaturated fatty acids are changed from nonconjugated to conjugated double bonds. This process takes place almost immediately after hydroperoxides have been formed. As compared to peroxide value determination, the measurement of conjugated dienes is much faster and simpler and does not depend on chemical reactions (Senanayake, 2013). CD was increased with increasing time of frying (Figure 2). Formation of conjugated diene in canola oil containing BHA and *Teucrium polium* extract was lower than control. The results showed no significant difference among CD values of oil treated with different concentration of *Teucrium polium* extract. So, CD was not meaningfully affected by these concentrations of extract. The CD in control was highest among the samples at the end of frying.

#### 3.2.7 Total polar content

The content of total polar compounds in used deep fat frying oil is until today an important criterion for assessing the decrease of fat quality. TPC is considered to be nonvolatile compounds of fat a higher polarity than triacylglycerols, resulting from thermal hydrolytic and oxidative alteration (Marinova et al., 2012). The total polar content increased with increasing frying time for all of samples, and a sharp rise was observed in 30 hr of frying (Table 5). The total polar content for BHA and *Teucrium polium* extract did not reach the maximum level of allowed in frying oil according to regulations in many countries (25%) during 30 hr frying but TPC of control sample reaches 25% after 27 hr. TPC for the control sample was higher than BHA and sample treated with extracts but BHA showed the better ability to reduction of total polar content after 30 hr of frying. The enhanced rates of TPC for oil treated with different concentration of *Teucrium polium* extract were found to be 16.14, 17.08, and 18.57% for 1,000, 600, and 200 ppm, respectively. As shown by the result, increase in total polar content was dependent on extract concentration.

**Table 5:** Total polar content (%) of canola oil with different concentrations of *Teucrium polium* extract (ppm) during frying process at 180°C

	Time (h)						
	0	5	10	15	20	25	30
200 ppm	2.67 ± 0.41 ^Db^	2.78 ± 0.43 ^Db^	3.11 ± 0.48 ^Cb^	3.22 ± 0.50 ^Cb^	2.79 ± 0.43 ^Db^	3.80 ± 0.58 ^Bb^	18.57 ± 2.88 ^Ab^
600 ppm	2.46 ± 0.37 ^Db^	2.56 ± 0.39 ^Db^	2.86 ± 0.44 ^Cc^	2.97 ± 0.46 ^Cb^	2.57 ± 0.39 ^Db^	3.50 ± 0.54 ^Bb^	17.08 ± 2.65 ^Ab^
1,000 ppm	2.32 ± 0.35 ^Db^	2.42 ± 0.37 ^Db^	2.71 ± 0.42 ^Cc^	2.80 ± 0.43 ^Cb^	2.43 ± 0.37 ^Db^	3.31 ± 0.51 ^Bb^	16.14 ± 2.50 ^Ac^
BHA	2.15 ± 0.33 ^Db^	2.19 ± 0.34 ^Db^	2.45 ± 0.38 ^Cc^	2.54 ± 0.39 ^Cb^	2.20 ± 0.34 ^Cb^	3.00 ± 0.46 ^Bb^	14.63 ± 2.27 ^Ad^
Control	3.98 ± 0.61 ^Da^	4.38 ± 0.68 ^Da^	4.74 ± 0.73 ^Da^	4.65 ± 0.71 ^Da^	6.06 ± 0.94 ^Ca^	19.59 ± 3.02 ^Ba^	37.83 ± 5.87 ^Aa^

Note

Means ± standard error within a row with the same uppercase letters is not significantly different at *p* < .05. Means ± standard error within a column with the same lowercase letters is not significantly different at *p* < .05.

#### 3.2.8 Color changes

**Figure 3:**
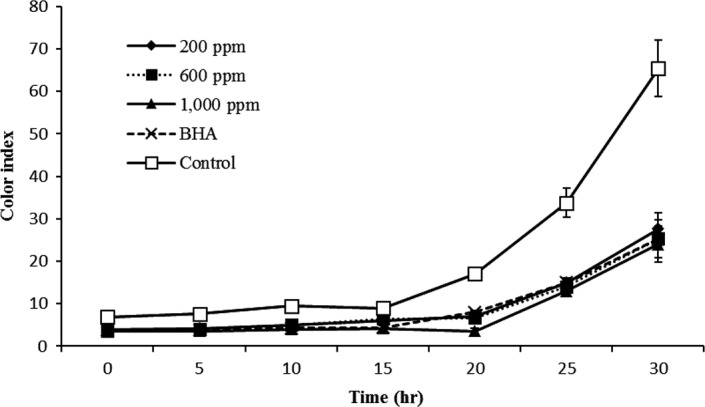
Changes in color index of canola oil with different concentrations of *Teucrium polium* extract (ppm) during frying process at 180°C

Color is a physical parameter widely used to evaluate rancidity development of commercial and domestic purposes. The color changes of oil during heating are related to the chemical decomposition and formation of oxidation products such as hydroperoxides, conjugated dienoic acids, ketones, and hydroxides (Maleki, Ariaii, & Fallah, 2016). The color of all samples was gradually increased with increasing frying cycle, but a considerable increase in 25 hr for extract and BHA and 20 hr of frying for control was observed. Color of control sample was significant (*p* < .05) higher than other sample and oil treated with 1,000 ppm of *Teucrium polium* extract had a lower color after 30 hr of frying (Figure 3). Increasing trend of BHA was similar to different concentration of *Teucrium polium* extract. Result shows that BHA and *Teucrium polium* extract can decrease the color due to degradation of prolonged frying oil.

## 4 CONCLUSION

Phenolic compounds are widely distributed in nature and according to this paper, *Teucrium polium* is a natural source of phenolic compounds. *Teucrium polium* ultrasound‐assisted extract is qualified to delay canola oil oxidation throughout its use in frying. This may be due to the presence of tocopherols and phenolic compounds such as flavonoids and tannins in the extract. Canola oil formulated with *Teucrium polium* extract showed the lower degradation of oil oxidation products during frying. Thus, the acidity, peroxide value, carbonyl value, polar content, conjugated diene, and color index were decreased, but oil stability index and iodine value were increased. Consequently, *Teucrium polium* extract can be used as natural antioxidant to stability and safety of canola oil during frying.

## CONFLICT OF INTERESTS

The authors have declared no conflict of interest.

## ETHICAL APPROVAL

This study does not involve any human or animal testing.
